# UV LED disinfection as a novel treatment for common salmonid pathogens

**DOI:** 10.1038/s41598-024-79347-6

**Published:** 2024-11-18

**Authors:** Kyle D. Rauch, Jessica L. Bennett, Amina K. Stoddart, Graham A. Gagnon

**Affiliations:** Department of Civil and Resource Engineering, Centre for Water Resources Studies, Dalhousie University, 1360 Barrington Street, Halifax, B3H 4R2 NS Canada

**Keywords:** UV LEDs, Aquaculture, Disinfection, Water Treatment, *Aeromonas salmonicida*, *Yersinia ruckeri*, Engineering, Civil engineering

## Abstract

*Aeromonas salmonicida* and *Yersinia ruckeri* are common pathogenic bacteria that impact salmonid aquaculture. Although vaccinations are available against both organisms, large-scale vaccination efforts can be expensive, cumbersome, and are not always reliable. Alternatively, these pathogens have been effectively inactivated using UV radiation from mercury-based systems. These systems are energy intensive and fragile which currently limits their use to closed and semi-closed production systems. UV light emitting diodes (UV LEDs) have recently emerged as a novel alternative to traditional mercury-based treatment. UV LEDs have durable housing, a relatively low energy draw, can be powered by a battery source and are adaptable to challenging environments. This study examined the effectiveness of three UV LED wavelengths for disinfection of *A. salmonicida* and *Y. ruckeri* in pure culture and resuspended in a wastewater matrix. All tested UV LEDs were effective in disinfecting both organisms. 267 and 279 nm wavelengths outperformed 255 nm disinfection in both test matrices. Particulate matter from wastewater reduced the upper limit of treatment for *A. salmonicida* but results still indicated that all wavelengths were effective for disinfection in a challenging matrix. This study represents the first use of UV LEDs for disinfection of *A. salmonicida* and *Y. ruckeri* and provides impact to aquaculture producers looking to implement novel technologies for disease control.

## Introduction

As the global human population continues to rise, the acquisition of reliable, safe and sustainable food sources is integral to ensuring international food security. The seafood industry is a significant contributor to global food supply with production reaching an all-time high of 179-million tons (valued at 401 billion USD) in 2018^1^. Though global fish consumption has increased by an average of 3.1% annually since 1961, commercial fisheries landings have remained relatively stagnant for the last three decades. Conversely, aquaculture production has risen by 7.5% per year since 1970 and has become fundamental in bridging the gap between seafood demand and the limitations of capture fisheries.

Pathogenic infections among cultured organisms are one of the foremost challenges facing aquaculture producers. Growing seafood demand has resulted in the use of progressively intensive culture methods to bolster revenue and product yield. High stocking densities (and concurrently, increased loading of high nutrient artificial feeds) can result in inadequate water quality conditions^2^, increased animal stress^3^ and elevated proliferation of infectious disease^4,5^. Disease among cultured cohorts not only has significant impacts on overall production, but also on food safety, environmental security, and human health^6^.

*Aeromonas salmonicida* and *Yersinia ruckeri* are two common gram-negative bacteria that affect the aquaculture industry and are responsible for considerable economic losses^7,8^. Maldonado-Miranda et al. estimated that pathogenic infection by *Yersinia ruckeri* and *Aeromonas* genera contribute to approximately 120 and 540 million dollars/year in lost global revenue, respectively^9^. Infection by these agents causes furunculosis (*A. salmonicida*) and enteric red mouth disease (*Y. ruckeri*), both of which can result in high levels of morbidity and mortality in affected cohorts. The most common protection measure against these organisms is preventative vaccination. Previous work has indicated that immune responses to *A. salmonicida* vaccination are variable^10^; while reducing mortality, vaccination does not always prevent infection. Vaccination efforts against *Y. ruckeri* seem to be more reliable^11^ but require regular vaccination programming or “boosters” to remain effective^12^. Large-scale vaccination regimes are also expensive, time consuming and increase animal stress which may heighten susceptibility to disease^13^. Moreover, the incidence of antibiotic-resistant strains of both *A. salmonicida*^14^ and *Y. ruckeri*^15^ further complicates treatment efforts and highlights the need for alternative mitigation measures.

UV treatment is a common disinfection technique used throughout closed and semi-closed aquaculture systems^16,17^. Photons within the UV-C spectrum (200–280 nm) can effectively denature the DNA of microorganisms, rendering them harmless and unable to replicate. Low-pressure UV lamps, which are monochromatic at 254 nm, are the most commonly used lamp type in the aquaculture industry. Medium-pressure lamps, which are polychromatic within the 200–400 nm range, are more seldomly utilized due to their higher operating costs and relative inefficiency. Previous work using a low-pressure 254 nm treatment system noted that a marked (4-log) reduction in both *A. salmonicida* and *Y. ruckeri* can be achieved at UV doses of 5.9 and 4.0 mJ cm^− 2^, respectively^18^. However, traditional treatment systems (i.e., low-pressure and medium-pressure lamps) use fragile mercury-based bulbs which often have long warm-up times and are energy inefficient. This currently limits the use of traditional UV treatment systems to land-based aquaculture systems.

UV LED-based treatment technologies offer a novel alternative to the traditional mercury systems that are currently considered the aquaculture industry standard. UV LEDs are made of mercury-free semiconductors encased in durable quartz housing and can be powered by a battery source. Though wall-plug efficiencies of UV LEDs currently remain lower than traditional low-pressure mercury-based systems (< 10% and 30–35%, respectively^19^), UV LEDs can be densely packed to deliver highly efficient disinfection. A full-scale demonstration of UV LEDs at a municipal wastewater facility showed that high-density UV LEDs provided highly efficient disinfection of *E. coli*^20^. Further, UV LEDs are mercury free and would be compliant with mercury regulations for manufactured lamps such as Regulation (EU) 2024/1849 of the European Parliament^21^. UV LEDs can also be tailored to emit radiation at specific wavelengths within the UV spectrum which allows for more efficient and targeted disinfection of microorganisms^22^. Moreover, the unique modular nature of UV LEDs offers greater versatility and wider range of application compared to traditional UV disinfection systems. Though select work has explored the use of UV LEDs for disinfection within closed aquaculture systems^23,24^, research in this area has been limited.

This study establishes the effective use of UV LED-based disinfection as an alternative mitigation technology for two pathogens of significant interest to the aquaculture industry, *Aeromonas salmonicida* and *Yersinia ruckeri*. Several UV LED wavelengths were tested (λ = 255, 267 and 279 nm) to determine their efficacy for inactivation of both target organisms in pure cultures and suspended in a municipal wastewater effluent matrix. To the author’s knowledge, this study demonstrates the first instance of UV LED disinfection of these bacteria and provides an important application of technology development for the aquaculture industry. Moreover, this work provides impact to not only recirculating aquaculture system users looking to improve disinfection efficiency and reduce energy cost, but also to other stakeholders in aquaculture and marine industries interested in novel disinfection alternatives or supplemental protection measures to compliment conventional disease prevention techniques.

## Materials and methods

### Microbial methods

*A. salmonicida* (ATCC 33658) and *Y. ruckeri* (ATCC 29475) were sourced from Cedar Lane (Ontario, Canada) and glycerol stocks were prepared from freeze dried pellets as per the manufacturer’s instructions. Glycerol stocks were stored at -80 °C. All agars, media, and glassware were autoclaved for 15 min at 121 °C to ensure sterility (AMSCO Lab 250, Steris Co, United Kingdom). Phosphate buffered saline (PBS) solution, used for cell cleaning and dilution media, was prepared in accordance with *Standard Methods for the Examination of Water and Wastewater*^25^.

Working solutions were prepared by first inoculating 9.9 ml of fresh tryptic soy broth (TSB; Difaco, USA) with 100 µL of glycerol stock and incubating overnight at 20 °C and 180 RPM. The following day, 1 mL of the overnight culture was used to inoculate 9 mL of fresh TSB and incubated at 20 °C and 180 RPM until the mid- to late- exponential phase was reached. For *A. salmonicida* and *Y. ruckeri*, this was approximately 18 and 6 h, respectively. Growth phase and concentration were confirmed each experimental day with OD600 readings using a DR5000 spectrometer (HACH, USA). Cells were then pelleted by centrifuging for 10 min at 3000 RPM. The supernatant was then decanted, and fresh PBS solution was used to resuspend and rinse the cells at 3000 RPM for 1 min. Pelleting and rinsing were completed three times before finally spiking the bacteria in fresh PBS at an approximate final concentration of 10^7^ CFU/mL.

Both species were enumerated on tryptic soy agar plates. Samples were 10-fold serially diluted in sterile PBS to ensure the concentration was within detectable limits. A flame sterilized glass rod was used to spread 0.1 mL of sample across the face of the agar plate. Plates were wrapped in tinfoil and stored in a dark cupboard at room temperature for 3–5 days. Plates containing 0-300 colonies were considered for evaluation.

### Wastewater preparation

An enhanced primary municipal wastewater effluent was used as a surrogate wastewater matrix in place of aquaculture process water as they share similarities of higher particulate and organics loading. Samples were collected from a chemically enhanced primary wastewater treatment facility in Halifax Nova Scotia, Canada with average daily flows of 134 m^3^/d, total suspended solids (TSS) < 40 mg/L, biological oxygen demand (BOD) < 50 mg/L and typical conductivity of 2–3 ms/cm. Wastewater within the treatment plant undergoes coarse and fine screening, aerated grit removal, high-rate clarification using a Densadeg system, and UV disinfection before being discharged to the environment.

Samples were collected just prior to the inlet of the UV system at the plant. Samples were then autoclaved at 121 °C for 15 min for sterilization. Following sterilization, a portion of the autoclaved wastewater effluent was plated on the same non-selective agar as the bacterial suspensions to ensure there was no residual bacterial contamination related to the matrix being used. Negative controls were also included in in each set of experimental replicates to ensure that there was no external bacterial contamination All negative controls showed no growth, indicating effective sterilization of the wastewater matrix. The autoclaved effluent was stored at -20 °C immediately after sterilization until thawing the day before experimentation. Pathogen species were then added to water samples after propagation in the same manner as the previous working solutions to have a final concentration of 10^7^ CFU/mL. Both *A. salmonicida* and *Y. ruckeri* demonstrate high UV sensitivity; accordingly, this concentration was chosen to obtain robust and representative kinetics curves for each species. Samples were shaken for an additional 1.5 h at 20 °C and 180 RPM to allow for adequate bacterial attachment to particulate matter in the sample.

Total suspended solids (TSS), UVT%, total iron, and ferrous iron were measured as these parameters can hinder UV disinfection. TSS was measured according to the *Standard Methods for the Examination of Water and Wastewater* Method 2540D (APHA, AWWA, and WEF 2012). 200 mL of wastewater was filtered through a precleaned, dried, and weighed glass microfibre filter using a vacuum pump apparatus. The filters were dried at 105 °C for at least 1 h following filtration before determining the total weight. UVT% and iron (total and ferrous) were measured on a DR5000 spectrometer (HACH, USA). UVT% was measured using a 1 cm pathlength quartz cuvette and collected at each UV LED peak wavelength. Ferric iron concentrations were estimated by taking the difference of total and ferrous iron concentrations.

The TSS of the original wastewater effluent was 12.5 mg/L. The total iron and ferrous iron concentrations were 0.30 mg/L and 0.04 mg/L, respectively, which indicates that ferric iron concentrations were approximately 0.26 mg/L. The UVT% was low with all samples at all wavelengths having UVT% less than 50% with longer wavelengths having higher UVT% (Table 1).

**Table 1:** UVT% values for *A. Salmonicida* and *Y. ruckeri* suspended in municipal wastewater effluent at three wavelengths. Mean(SD). *N* = 3.

Wavelength	255 nm	267 nm	279 nm
***Y. ruckeri***	31.9 (1.9)	34.5 (2.1)	40.6 (2.3)
***A. salmonicida***	37.6 (0.4)	41.3 (1)	47.4 (0.4)

### Fluence determination

Initial irradiance measurements were collected with a USB4000 spectroradiometer and SpectraSuite software (Ocean Optics Inc., FL, USA). Measurements were taken 4 cm from the edge and in the center of the collimator tube. The measured irradiance (I_0_) was then corrected to account for the non-uniform irradiance profile, optics, and contaminate using factors described by Bolton and Linden^26^. The average irradiance was calculated using Eqs. 1 & 2.

1 $$\:{\text{I}}_{\text{c}\text{o}\text{r}}={\text{I}}_{0}\:\times\:\:\text{P}.\text{F}.\:\:\times\:\:\text{D}.\text{F}.\:\:\times\:\text{R}.\text{F}.\:\:\times\:\text{W}.\text{F}.$$

2 $$W.F. = (1 - {10^{ - A1cm \times l}})/(2.303 \times {A_{1cm}} \times l)$$

Where P.F. is the petri factor, D.F. is the divergence factor, R.F. is the reflectance factor (0.975), W.F. is the water factor, A_1cm_ is the absorbance for a 1 cm pathlength at the peak wavelength of the UV LED being used, and l is the pathlength of the sample being treated in cm. Exposure times were then determined by dividing the required fluence by the corrected irradiance. Relevant parameters used to calculate UV fluences and range of associated exposure times are given in Table 2 below.

**Table 2:** Factors used in the calculation of UV fluences for 255, 267, and 279 nm UV LEDs, UV fluences, and Exposure Times Associated with UV fluences.

	Wavelength		
	255 nm	267 nm	279 nm
***Petri Factor***	0.891	0.857	0.846
***Incident Irradiance (mW/cm*** ^***2***^ ***)***	0.088	0.404	0.511
***UV Fluences (mJ/cm*** ^***2***^ ***)***	1–10	1–10	1–10
***Exposure Times (Pure Water; min)***	0.13–2.3	0.03–0.53	0.02–0.42
***Exposure Times (Wastewater; min)***	0.37–3.75	0.08–0.85	0.07–0.63

### Experimental

A collimated beam apparatus (Pearl Beam Aqua, Aquisense Technologies Limited, Kentucky, USA) equipped with UV LEDs with peak wavelength emissions of 255, 267, and 279 nm was used for this study. The system was arranged with the edge of the collimator 4 cm from the surface of the sample. Fluences between 0 and 10 mJ/cm^2^ were used to develop UV fluence response curves for both species at all wavelengths. 26 mL of sample volume was used throughout experiments. A 6 cm diameter glass petri dish was used as the sample container. Samples were mixed for a minimum of 5 s prior to starting exposure. Following treatment, samples were enumerated using the standard plate counting method previously described and an untreated sample was enumerated as a positive control. Only plates containing 1–300 CFU/mL were quantified. Plates containing < 1 CFU/mL were counted as zero. All exposures and plating were completed under subdued red light to ensure effects of photoreactivation were minimized. All samples were collected as full experimental triplicates.

### Statistical analysis

Linear and non-linear models were fitted using the lm() and nls() functions in R 4.0.3^27^, respectively. The linear models (Eq. 3) were only fit through the log linear region of the observed data which was determined visually.

3 $$\:{N}_{D}\:=\:{N}_{0}{10}^{-kD}\:$$

Where N_D_ is the concentration of the population after UV dose, D, N_0_ is the initial concentration of the population, and k is the inactivation rate constant defined by the linear slope of the curve on a base 10 logarithmic scale. The non-linear models used a modified form of the heat inactivation model presented by Geeraerd^28^ that only includes the log linear and shoulder phases (Eq. 4).

4 $$\:{N}_{t\:}=\:({N}_{0}-{N}_{res}){10}^{-kD}+{N}_{res}$$

Where N_res_ is the concentration of a resistant population. The goodness of fit of the models were evaluated using the R^2^ or RMSE for the linear and non-linear models, respectively.

## Results and discussion

### Inactivation performance

**Fig. 1:**
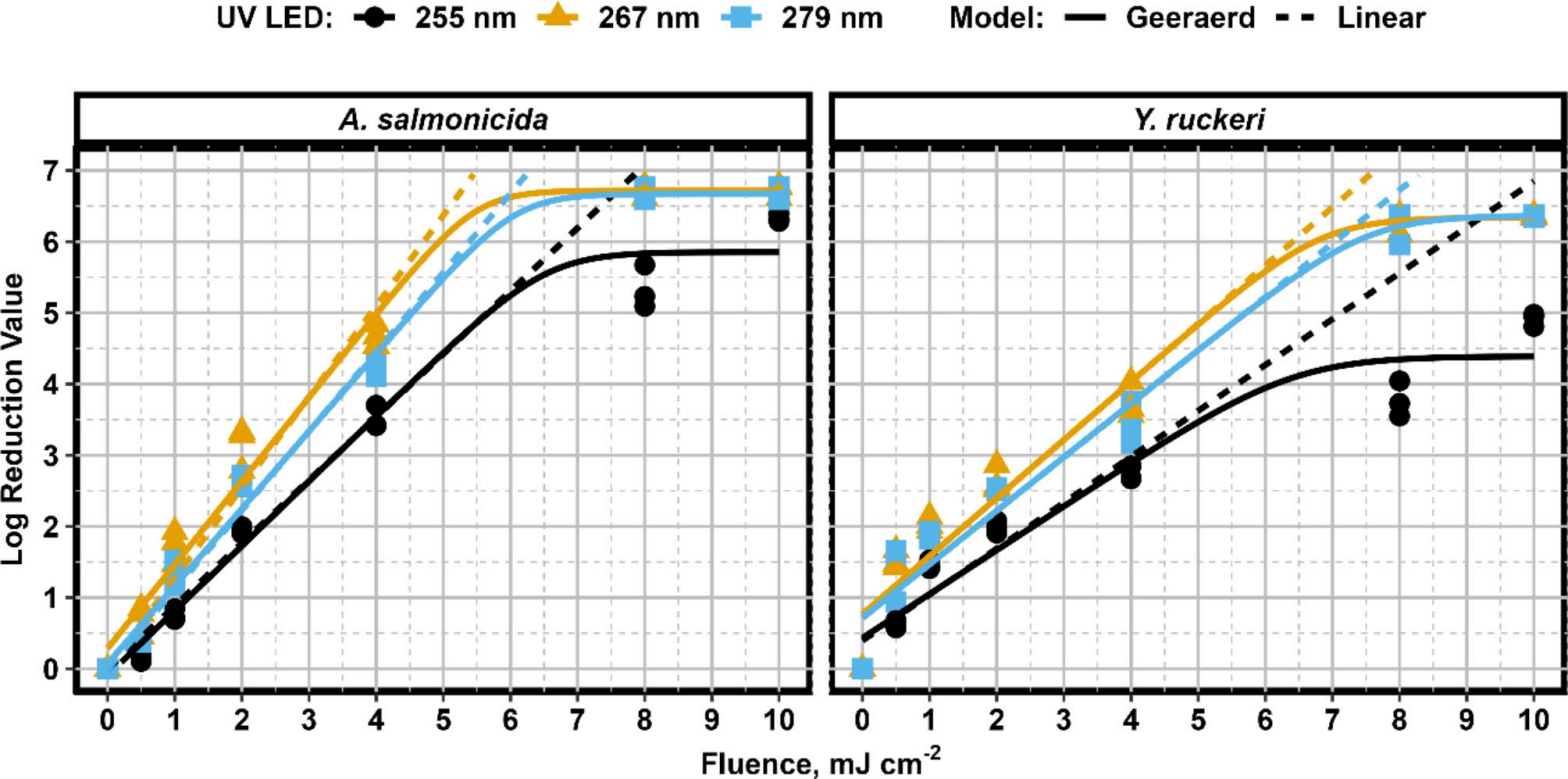
Inactivation kinetics for pure culture suspensions of *A. salmonicida* and *Y. ruckeri* at 255, 267, and 279 nm wavelengths.

Disinfection of *A. salmonicida* and *Y. ruckeri* resuspended in PBS was found to be species and wavelength dependant (Fig. 1). No substantial difference in performance was observed between the 267 and 279 nm wavelengths for both species. Near complete inactivation (~ 6.7 log) of *A. salmonicida* was observed at a fluence of 8 mJ/cm^2^. A similar result of complete inactivation (~ 6.4 log) of *Y. ruckeri* was achieved at a fluence of 10 mJ/cm^2^. These data suggest that the observed shoulder phase in the kinetics for the 267 and 279 nm UV LEDs is an artefact related to the detection limit. The 255 nm UV LED treatment produced lower levels of disinfection, with *A. salmonicida* being more responsive than *Y. ruckeri.* The 255 nm treatments at 10 mJ/cm^2^ produced a 6.33 ± 0.06 and 4.91 ± 0.09 log reduction for *A. salmonicida* and *Y. ruckeri*, respectively. This log reduction produced complete disinfection (i.e., no colony growth on the agar plates for undiluted samples) for *A. salmonicida*. The markedly lower log reduction observed in *Y. ruckeri* samples and the presence of a shoulder phase in a pure culture indicated that this pathogen may have an increased resistance to 255 nm wavelengths. Overall, the pure culture work indicated that UV LEDs tuned at either 267–279 nm provided optimized treatment for disinfection of either of these pathogens in a clean matrix.

Liltved and Landfald used a mercury-based system to examine inactivation kinetics at 254 nm for both species. The authors observed a 3-log reduction at a fluence of 3.2 and 1.2 mJ/cm^2^ for *A. salmonicida* and *Y. ruckeri*, respectively^29^. Based on the kinetics of this study, similar log reductions for these pathogens would be achieved with 255 nm UV LEDs at fluences of 3.3 and 4 mJ/cm^2^. The substantial difference in performance for *Y. ruckeri* could potentially be due to differences in the UV characterization methods used. The standard method for UV light characterization adopted today had not been developed at the time of the previous study and includes many corrections factors that could not be applied in previous work^26^.

Using alternative wavelengths available only to UV LEDs allows for tailored disinfection and was observed to substantially reduce the required UV dose, and thus the energy for treatment. For example, a 50% reduction in fluence (8 to 4 mJ/cm^2^) was required to achieve roughly the same level of log inactivation for *Y. ruckeri* if either the 267–279 nm UV LED is used instead of the 255 nm. This notable increase in germicidal efficiency gained from selecting optimized wavelengths is a known feature of UV LEDs as each microorganism will have a unique action spectra (relative response to different wavelengths) with a peak germicidal wavelength between 240 and 280 nm^30^. Taking advantage of the action spectra of a microorganism can increase the feasibility of treatment as the increase in germicidal efficiency offsets the current energy efficiency limitations experienced by UV LEDs. To the authors knowledge, this is the first set of results showing optimized wavelengths for *A. salmonicida* and *Y. ruckeri* and provides further evidence for the applicability of UV LEDs for treatment in the aquaculture sector.

### Impacts of particulate loading and iron

**Fig. 2:**
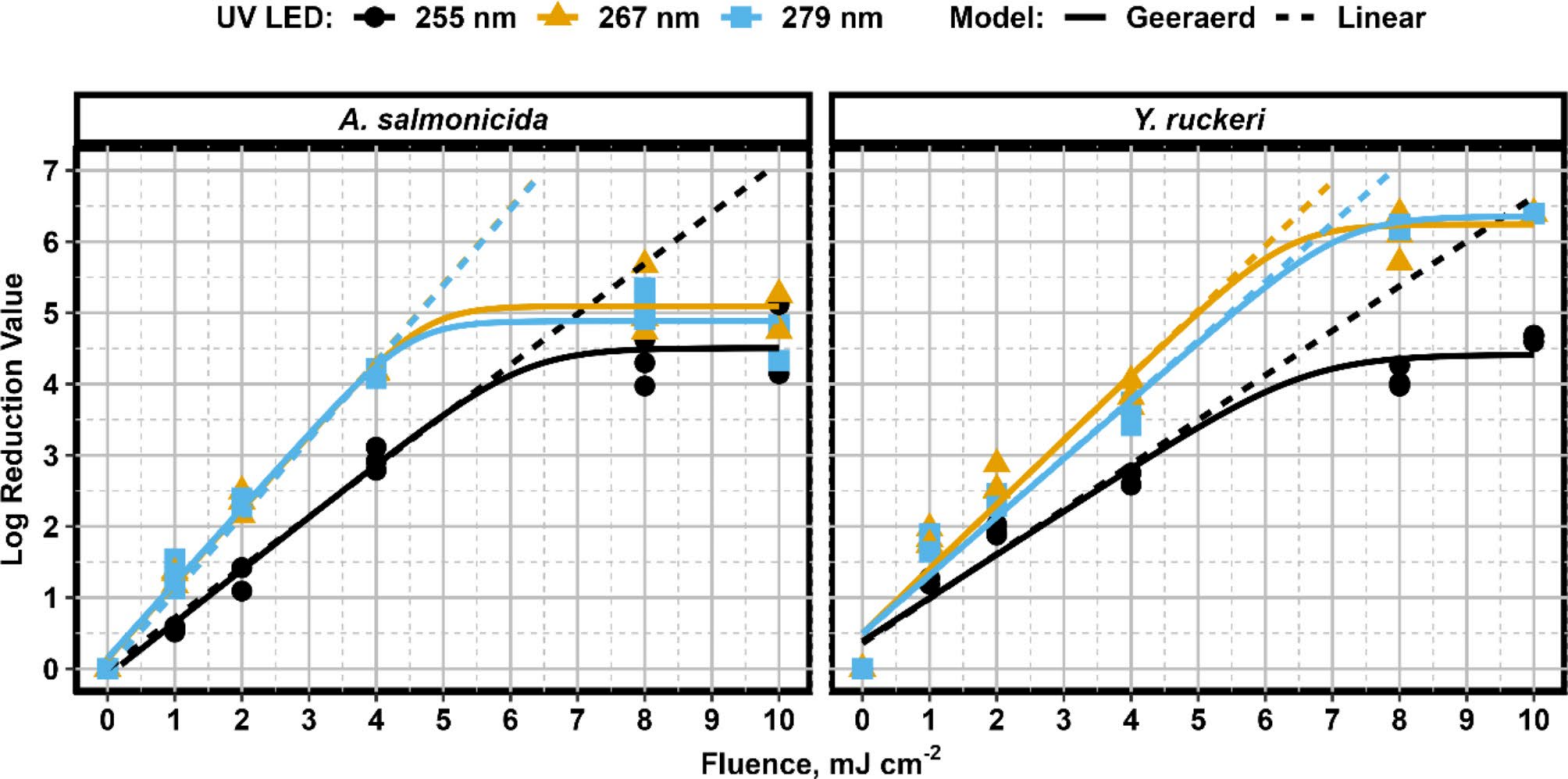
Inactivation kinetics for *A. salmonicida* and *Y. ruckeri* cultures suspended in municipal wastewater effluent at 255, 267, and 279 nm wavelengths.

Aquaculture process waters are often heavily loaded with particulate and organic matter which may significantly impact UV disinfection. As such, tests were repeated in a municipal wastewater matrix to understand the impacts of these variables on treatment performance. A similar overall trend between wavelengths was observed when *Y. ruckeri* and *A. salmonicida* were suspended in wastewater effluent (Fig. 2) versus in pure culture. *A. salmonicida* was noted to have a shoulder phase above a UV dose of 4 mJ/cm^2^ for all wavelengths in wastewater whereas a shoulder phase was not observed until a UV dose of 8 mJ/cm^2^ in pure culture. The earlier shoulder phase and incomplete disinfection observed for *A. salmonicida* in the wastewater matrix may have been the result of particle shielding. In contrast, no substantial change in disinfection kinetics occurred for *Y. ruckeri*. This may indicate that *Y. ruckeri* has a lower affinity for particulate attachment than *A. salmonicida*.

The non-linear kinetics observed for the *Y. ruckeri* suspensions include a shouldering phase for the 255 nm UV LED treatment in both the pure culture and wastewater matrix. This finding indicates that either aggregation of cells was occurring^31^, or that a UV-resistant sub-population was present^32,33^, resulting in incomplete inactivation. UV resistant sub populations in monocultured *E. coli* suspensions are rare but have been shown to exist as a stochastic artefact and not as a product of genetic mutation in response to UV treatement^33^. While the pure *Y. ruckeri* suspension was developed from a monoculture, the substantial tolerance to UV treatment suggests that aggregation of cells is the more plausible explanation for the deviation from linear kinetics. Earlier work has indicated that UV treatment may cause cell aggregation^31,34^, and it has been suggested that this mechanism is both intensity and wavelength dependant^34^. Previous studies have only observed UV induced aggregation occurring at 254 nm, which may explain why only the 255 nm UV LED kinetics produced a shouldering phase.

Shouldering phases were present in all inactivation kinetics for *A. salmonicida* and N_res_ (upper limit of treatment) values were greater for 267 and 279 nm wavelengths than for 255 nm. Increased N_res_ values may be related to the water quality of the wastewater matrix. The increase in UVT% at the longer wavelengths may allow for better penetration of UV radiation into the sample; however, differences in UVT% are accounted for when calculating the fluences and should have limited impact on treatment. Iron typically is found in a ferric particulate form at a near-neutral pH and can complex with organic matter^35^. This complex results in the formation of larger particulate that provides shaded regions which may protect the microorganism and scavenge available UV energy. Furthermore, high UV absorbing particles have been shown to provide more protection from treatment compared to non-UV absorbing particles^36^. The larger N_res_ values recorded for the 267 and 279 nm treatments for *A. salmonicida* may suggest that the particulate matter had a lower propensity to absorb UV light at these wavelengths and thus provided less protection to the attached microorganisms.

### Summary of modeling kinetics

Two modeling approaches (Linear and Geeraerd) were applied to determine the inactivation rate constant in the log linear region for both species suspended in both matrices. The use of Geeraerd’s model for sigmoidal data accurately describes the inactivation rate constant while removing any potential error from visually determining the log linear region^37^. A comparison of key parameters from both models (Table 3) showed agreement between modeled inactivation rate constants. *A. salmonicida* was found to be more susceptible to UV disinfection in comparison to *Y. ruckeri* at all wavelengths tested. In PBS suspension, inactivation rate constants were found to be 47.2, 44.4, and 48.5% higher for *A. salmonicida* at 255, 267, and 279 nm, respectively. This effect was less prominent in the wastewater suspension where only a 21.7, 16.0, and 28.4% increase in the inactivation rate constant for *A. salmonicida* was found for the 255, 267, and 279 nm wavelengths, respectively. Wavelengths of 267 and 279 nm were observed to produce higher inactivation rates compared to 255 nm for all suspensions tested, with 267 nm performing slightly better compared to the 279 nm UV LED. This is consistent what has been found for other bacterial species where the action spectra peaks at wavelengths other than 255 nm^30^. Furthermore, comparing the inactivation rate constants of the microorganisms in the pure culture to the wastewater matrix there was no substantial change in all cases except for *A. salmonicida* at 255 nm (Δ-0.169 cm^2^/mJ) and *Y. ruckeri* at 267 nm (Δ + 0.088 cm^2^/mJ) and 279 nm (Δ + 0.084 cm^2^/mJ). However, the 95% confidence intervals for these differences indicates no statistical significance.

**Table 3:** Summary of key modelling parameters for *Y. Ruckeri* and *A. Salmonicida* in pure culture and municipal wastewater effluent from applied Linear and Geeraerd models.

Species	Matrix	LED, nm	k_nonlinear_, cm^2^/mJ	*N*_res_, LRV	RMSE	Reason for *N*_res_	k_linear_, cm^2^/mJ	*R* ^2^
***AS***	**Pure**	255	0.910 (0.780–1.04)	5.85 (5.56–6.15)	0.312	Detection Limit	0.885 (0.834–0.935)	0.990
		267	1.18 (1.05–1.30)	6.72 (6.43–7.01)	0.309	Detection Limit	1.27 (1.16–1.39)	0.976
		279	1.09 (0.991–1.19)	6.67 (6.45–6.89)	0.235	Detection Limit	1.13 (1.04–1.19)	0.985
***YR***		255	0.618 (0.447–0.789)	4.39 (3.99–4.79)	0.407	Aggregation/Artefact	0.645 (0.510–0.780)	0.892
		267	0.817 (0.650–0.983)	6.34 (5.94–6.74)	0.405	Detection Limit	0.817 (0.615–1.02)	0.855
		279	0.734 (0.602–0.905)	6.36 (5.90–6.74)	0.404	Detection Limit	0.751 (0.552–0.951)	0.836
***AS***	**WW**	255	0.741 (0.627–0.856)	4.51 (4.26–4.75)	0.249	Particle Shielding	0.712 (0.669–0.754)	0.992
		267	1.05 (0.937–1.61)	5.09 (4.88–5.31)	0.223	Particle Shielding	1.08 (1.03–1.13)	0.995
		279	1.05 (0.931–1.17)	4.88 (4.67–5.10)	0.225	Particle Shielding	1.08 (1.02–1.14)	0.993
***YR***		255	0.609 (0.475–0.743)	4.41 (4.11–4.72)	0.293	Aggregation/Artefact	0.627 (0.483–0.771)	0.904
		267	0.905 (0.737–1.07)	6.24 (5.89–6.60)	0.368	Detection Limit	0.907 (0.711-1.10)	0.913
		279	0.818 (0.673–0.963)	6.36 (6.00-6.60)	0.328	Detection Limit	0.824 (0.633–1.01)	0.903

AS = *A. salmonicida*; YR = *Y. ruckeri*; Bracketed values indicate 95% CI.

The upper threshold for treatment modeled as N_res_ was noted to be species, wavelength, and matrix dependant but was primarily an artifact related to the detection limit. Due to the high sensitivity of both species across all wavelengths, many of the testing conditions lead to complete disinfection at mid-range fluences. The model upper threshold for *A. salmonicida* was also significantly lower in the presence of particulate matter for all wavelengths. As discussed, the impacts of particulate matter on inactivation kinetics are well known to reduce the upper threshold of disinfection through particle shielding. This was not observed for *Y. ruckeri* as there was no significant or substantial difference in the modeled upper threshold between microorganisms suspended as a pure culture or in a wastewater matrix. Finally, 255 nm UV LED treatments were found to produce a significantly lower Nres value for *Y. ruckeri* in both pure and wastewater suspension in comparison to other wavelengths tested. In these circumstances complete disinfection was not observed, indicating some form of resistance. As previously noted, this is most likely due to UV-induced self-aggregation; however, further studies are required to confirm this.

## Conclusions

UV inactivation kinetics for *A. salmonicida* and *Y. ruckeri* were determined for three UV LED wavelengths. Results for both species showed that the 255, 267 and 279 nm UV LEDs were all effective at disinfecting both species, and wavelengths of 267 and 279 nm resulted in optimal treatment. Moreover, a shift to non-traditional UV wavelengths (i.e., those other than 254 nm) decreased the required UV dose by approximately 50% under certain conditions. These data show that UV LEDs can effectively disinfect the common salmonid pathogens, *A. salmonicida* and *Y. ruckeri*, even in challenging water quality conditions. As such, this treatment technology may be of interest for aquaculture producers looking to implement novel techniques to mitigate these pathogens. Furthermore, the adaptability of UV LEDs allows for versatility in system design and application which is currently limited by traditional mercury-based UV technology. Further, we have recently demonstrated the disinfection capabilities of 280 nm UV LEDs within a full-scale municipal wastewater facility and would expect similar performance in aquaculture systems^20^. Future work would benefit from additional testing of UV LEDs in pilot- or full-scale aquaculture systems to validate disinfection efficacy for real-world applications.

## Data Availability

The datasets generated and analyzed during the current study are available from the corresponding author on reasonable request.

## References

[1] *The State of World Fisheries and Aquaculture 2020*; FAO, (2020). 10.4060/ca9229en

[2] Xu, W. J., Morris, T. C. & Samocha, T. M. Effects of Two Commercial Feeds for Semi-Intensive and Hyper-Intensive Culture and Four C/N Ratios on Water Quality and Performance of Litopenaeus Vannamei Juveniles at High Density in Biofloc-Based, Zero-Exchange Outdoor Tanks. *Aquaculture 490*, 194–202. (2018). 10.1016/j.aquaculture.2018.02.028

[3] Kibenge, F. S. Emerging viruses in aquaculture. *Curr. Opin. Virol.* **34**, 97–103. 10.1016/j.coviro.2018.12.008 (2019). 30711892 10.1016/j.coviro.2018.12.008

[4] Rico, A. et al. Use of chemicals and Biological products in Asian aquaculture and their potential environmental risks: a critical review. *Rev. Aquac*. **4** (2), 75–93. 10.1111/j.1753-5131.2012.01062.x (2012).

[5] Wanja, D. W. et al. Fish Husbandry Practices and Water Quality in Central Kenya: Potential Risk Factors for Fish Mortality and Infectious Diseases. *Vet. Med. Int. 2020*, e6839354. (2020). 10.1155/2020/6839354 10.1155/2020/6839354 PMC7106927 32257096

[6] Stentiford, G. D. et al. New paradigms to help solve the global aquaculture Disease Crisis. *PLOS Pathog*. **13** (2), e1006160. 10.1371/journal.ppat.1006160 (2017). 28152043 10.1371/journal.ppat.1006160 PMC5289612

[7] Kumar, G., Menanteau-Ledouble, S., Saleh, M. & El-Matbouli, M. Yersinia Ruckeri, the Causative Agent of Enteric Redmouth Disease in Fish. *Vet. Res.* **46** (1), 103. 10.1186/s13567-015-0238-4 (2015). 26404907 10.1186/s13567-015-0238-4 PMC4581093

[8] Park, S. Y., Han, J. E., Kwon, H., Park*, S. C. & Hyung Kim*, J. Recent insights into Aeromonas Salmonicida and its bacteriophages in aquaculture: a Comprehensive Review. **30** (10), 1443–1457. (2020). 10.4014/jmb.2005.05040 10.4014/jmb.2005.05040 PMC9728264 32807762

[9] Maldonado-Miranda, J. J., Castillo-Pérez, L. J., Ponce-Hernández, A. & Carranza-Álvarez, C. Chapter 19 - summary of economic losses due to bacterial pathogens in Aquaculture Industry. In Bacterial Fish Diseases; (eds Dar, G. H., Bhat, R. A., Qadri, H., Al-Ghamdy, K. M. & Hakeem, K. R.) Academic, ; 399–417. 10.1016/B978-0-323-85624-9.00023-3. (2022).

[10] Škugor, S., Jørgensen, S. M., Gjerde, B. & Krasnov, A. Hepatic gene expression profiling reveals protective responses in Atlantic Salmon Vaccinated against Furunculosis. *BMC Genom.* **10** (1), 503. 10.1186/1471-2164-10-503 (2009). 10.1186/1471-2164-10-503 PMC2775754 19878563

[11] Villumsen, K. R., Neumann, L., Ohtani, M., Strøm, H. K. & Raida, M. K. Oral and anal vaccination confers full protection against Enteric Redmouth Disease (ERM) in Rainbow Trout. *PloS One*. **9** (4), e93845. 10.1371/journal.pone.0093845 (2014). 24705460 10.1371/journal.pone.0093845 PMC3976340

[12] Chettri, J. K. et al. Booster Immersion Vaccination using diluted Yersinia Ruckeri Bacterin confers Protection against ERM in Rainbow Trout. *Aquaculture*. **440**, 1–5. 10.1016/j.aquaculture.2015.01.027 (2015).

[13] Muktar, Y. & Tesfaye, S. Present Status and Future prospects of Fish Vaccination: a review. *J. Vet. Sci. Technol.* **07** (02). 10.4172/2157-7579.1000299 (2016).

[14] Trudel, M. V. et al. Diversity of antibiotic-resistance genes in Canadian isolates of Aeromonas Salmonicida Subsp. Salmonicida: dominance of pSN254b and Discovery of pAsa8. *Sci. Rep.* **6** (1), 35617. 10.1038/srep35617 (2016). 27752114 10.1038/srep35617 PMC5067588

[15] Orozova, P., Chikova, V. & Sirakov, I. *Full Length Res. Article* 8 .

[16] Kasai, H., Yoshimizu, M., Ezura, Y., Disinfection of Water & For Aquaculture. *Fish. Sci.* **68** (sup1), 821–824. 10.2331/fishsci.68.sup1_821. (2002).

[17] Rurangwa, E. & Verdegem, M. C. J. Microorganisms in Recirculating Aquaculture Systems and their management. *Rev. Aquac*. **7** (2), 117–130. 10.1111/raq.12057 (2015).

[18] Liltved, H. & Landfald, B. Influence of Liquid Holding Recovery and Photoreactivation on Survival of Ultraviolet-irradiated Fish pathogenic Bacteria. *Water Res.* **30** (5), 1109–1114. 10.1016/0043-1354(95)00276-6 (1996).

[19] A critical review of ultra-violet light emitting diodes as a one water disinfection technology Water Research X.** 25**, 100271. 10.1016/j.wroa.2024.100271 (2024). 10.1016/j.wroa.2024.100271 PMC11568360 39555045

[20] MacIsaac, S. A. et al. UV LED Wastewater Disinfection: the future is upon us. *Water Res. X*. **24**, 100236. 10.1016/j.wroa.2024.100236 (2024). 10.1016/j.wroa.2024.100236 PMC11637206 39669382

[21] European Union. *Regulation – 2024/1849 - EN - EUR-Lex*. https://eur-lex.europa.eu/eli/reg/2024/1849

[22] Beck, S. E. et al. Evaluating UV-C LED disinfection performance and investigating potential dual-wavelength synergy. *Water Res.* **109**, 207–216. 10.1016/j.watres.2016.11.024 (2017). 27889622 10.1016/j.watres.2016.11.024 PMC6145099

[23] Moreno-Andrés, J. et al. A comparison of Photolytic, photochemical and Photocatalytic Processes for Disinfection of Recirculation Aquaculture Systems (RAS) streams. *Water Res.* **181**, 115928. 10.1016/j.watres.2020.115928 (2020). 32504908 10.1016/j.watres.2020.115928

[24] Qi, W., Zhu, S., Shitu, A., Ye, Z. & Liu, D. Low concentration Peroxymonosulfate and UVA-LED combination for E. Coli Inactivation and Wastewater Disinfection from recirculating Aquaculture systems. *J. Water Process. Eng.* **36**, 101362. 10.1016/j.jwpe.2020.101362 (2020).

[25] APHA; AWWA; WEF. *Standard Methods for the Examination of Water and Wastewater* (District of Columbia, 2012).

[26] Bolton, J. R. & Linden, K. G. Standardization of methods for fluence (UV dose) determination in bench-scale UV experiments. *J. Environ. Eng.* **129** (March), 209–215. 10.1061/(ASCE)0733-9372(2003)129:3(209) (2003).

[27] R Core Team. R: A Language and Environment for Statistical Computing. (2020). https://www.R-project.org/

[28] Geeraerd, A. H., Herremans, C. H. & Impe, J. F. V. Structural model requirements to describe microbial inactivation during a mild heat treatment. **59**, 185–209. (2000). 10.1016/s0168-1605(00)00362-7 11020040

[29] Liltved, H. & Landfald, B. Influence of Liquid Holding Recovery and Photoreactivation on Survial of Ultraviolet-irradiated Fish pathogenic Bacteria. *Water Res.* **30** (5), 1109–1114 (1995).

[30] Beck, S. E., Wright, H. B., Hargy, T. M., Larason, T. C. & Linden, K. G. Action Spectra for Validation of Pathogen Disinfection in medium-pressure Ultraviolet (UV) systems. *Water Res.* **70**, 27–37. 10.1016/j.watres.2014.11.028 (2015). 25506761 10.1016/j.watres.2014.11.028

[31] Vitzilaiou, E., Kuria, A. M., Siegumfeldt, H., Rasmussen, M. A. & Knøchel, S. The impact of bacterial cell aggregation on UV inactivation kinetics. *Water Res.* **204**, 117593. 10.1016/j.watres.2021.117593 (2021). 34482094 10.1016/j.watres.2021.117593

[32] Cerf Tailing of Survival curves of bacterial spores. *J. OfApplied Bacteriol.* **42**, 1–19. 10.1111/j.1365-2672.1977.tb00665.x (1977). 10.1111/j.1365-2672.1977.tb00665.x 323208

[33] Ichikawa, S., Okazaki, M., Okamura, M., Nishimura, N. & Miyake, H. Rare UV-Resistant cells in clonal populations of Escherichia Coli. *J. Photochem. Photobiol B*. **231**, 112448. 10.1016/j.jphotobiol.2022.112448 (2022). 35490545 10.1016/j.jphotobiol.2022.112448

[34] Kollu, K. & Örmeci, B. UV-Induced Self-Aggregation of E. Coli after Low and medium pressure Ultraviolet Irradiation. *J. Photochem. Photobiol B*. **148**, 310–321. 10.1016/j.jphotobiol.2015.04.013 (2015). 26002538 10.1016/j.jphotobiol.2015.04.013

[35] Chen, J. & Browne, W. R. Photochemistry of Iron complexes. *Coord. Chem. Rev.* **374**, 15–35. 10.1016/j.ccr.2018.06.008 (2018).

[36] Templeton, M. R., Andrews, R. C. & Hofmann, R. Inactivation of particle-Associated viral surrogates by Ultraviolet Light. *Water Res.* **39** (15), 3487–3500. 10.1016/j.watres.2005.06.010 (2005). 16081130 10.1016/j.watres.2005.06.010

[37] Rauch, K. D., MacIsaac, S. A., Stoddart, A. K. & Gagnon, G. A. UV Disinfection Audit of Water Resource Recovery Facilities identifies System and Matrix limitations. *J. Water Process. Eng.* **50**, 103167. 10.1016/j.jwpe.2022.103167 (2022).

